# Piriformis Syndrome Masquerading as an Ischiofemoral Impingement

**DOI:** 10.7759/cureus.18023

**Published:** 2021-09-16

**Authors:** David P Newman, Liang Zhou

**Affiliations:** 1 Pain Management-Physiotherapy, Tripler Army Medical Center, Honolulu, USA; 2 Orthopaedic Surgery, Tripler Army Medical Center, Honolulu, USA

**Keywords:** piriformis syndrome, ischiofemoral impingement, deep gluteal pain syndrome, sacroiliac joint manipulation, piriformis stretching

## Abstract

Hip pain can have a number of different etiologies. Ischiofemoral impingement (IFI), an etiology causing extra-articular hip pain, shares many of the same symptoms as other causes of gluteal or inguinal pain, making its diagnosis difficult. We present a case of a young female with persistent deep gluteal pain who was diagnosed with IFI based on radiographic findings; however, a diagnostic injection into the quadratus femoris did not confirm IFI as the primary pain generator. The patient subsequently failed several trials of physical therapy designed to address this diagnosis. The diagnosis was expanded to include piriformis syndrome and the modified treatment approach resulted in complete resolution of her pain. The similarities of these pathologies resulted in a delay of definitive treatment and would have potentially required unnecessary surgery. This case study highlights the diagnostic conundrum clinicians face in the evaluation of gluteal hip pain and provides an algorithm for considering alternate diagnoses when conservative management fails to achieve expected results.

## Introduction

Ischiofemoral impingement (IFI) is a cause of extra-articular hip pain due to narrowing of the space between the ischial tuberosity and the lesser trochanter, resulting in entrapment of the quadratus femoris muscle. Radiographic evidence of IFI includes a decrease in the ischiofemoral and/or quadratus femoris spaces and intramuscular edema of the quadratus femoris muscle belly on magnetic resonance imaging (MRI) [1]. First described by Johnson in 1977 in patients following total hip arthroplasty, this diagnosis is becoming increasingly recognized as an underdiagnosed cause of hip pain [2,3].

Patients present clinically with gluteal and/or groin pain without an inciting event [4]. These symptoms are shared with a number of other intra- and extra-articular conditions to include femoroacetabular impingement, labral tears, low back pain, sacroiliac (SI) joint dysfunction, piriformis syndrome, cluneal nerve entrapment, snapping hip syndrome, psoas tendinitis, hamstring injuries, and gluteus minimus and medius tendinopathy. Shared symptomatology makes it very difficult to accurately diagnosis IFI syndrome, wherein the diagnosis may be missed or delayed following a prolonged course of failed conservative management.

We present a case of a young, healthy female with a several-year history of chronic, bilateral gluteal pain sustained after a long hike that was initially diagnosed as sciatica. MRI was consistent with IFI; however, a CT-guided injection into the quadratus femoris did not demonstrate the expected reduction in pain. While the patient was appropriately referred to physical therapy, she subsequently failed three courses of a structured program directed at treating presumed IFI. Based on these outcomes, and subsequent re-evaluation, the authors chose to adjust her rehabilitation program in line with piriformis syndrome. The purpose of this case study was to describe the application of a sequenced, impairment-based intervention directed at the SI joint and piriformis in a patient with presumptive IFI.

## Case presentation

A 24-year-old athletic female presented with a three-year history of chronic left posterolateral hip pain. The initial onset of pain occurred after a 16-mile hike carrying 45 pounds of weight on her back. Her initial pain was sharp in nature and radiated from the left posterolateral gluteal region down to her ankle. She also described intermittent numbness along the same distribution. There were no changes in bowel or bladder function or other red flag symptoms (i.e., saddle anesthesia, night pain, and unexplained weight loss). On presentation to a physician, her pain ranged from a 4/10 to 8/10 on the visual analog pain scale (VAS). A three-view lumbar series x-ray revealed mild degenerative osteoarthritis at the L3 through L5 facet joints. The SI joint and hip joint spaces were well maintained. The initial working diagnosis was left-sided sciatica, and she was subsequently referred to Orthopedics for a CT-guided injection.

**Figure 1:**
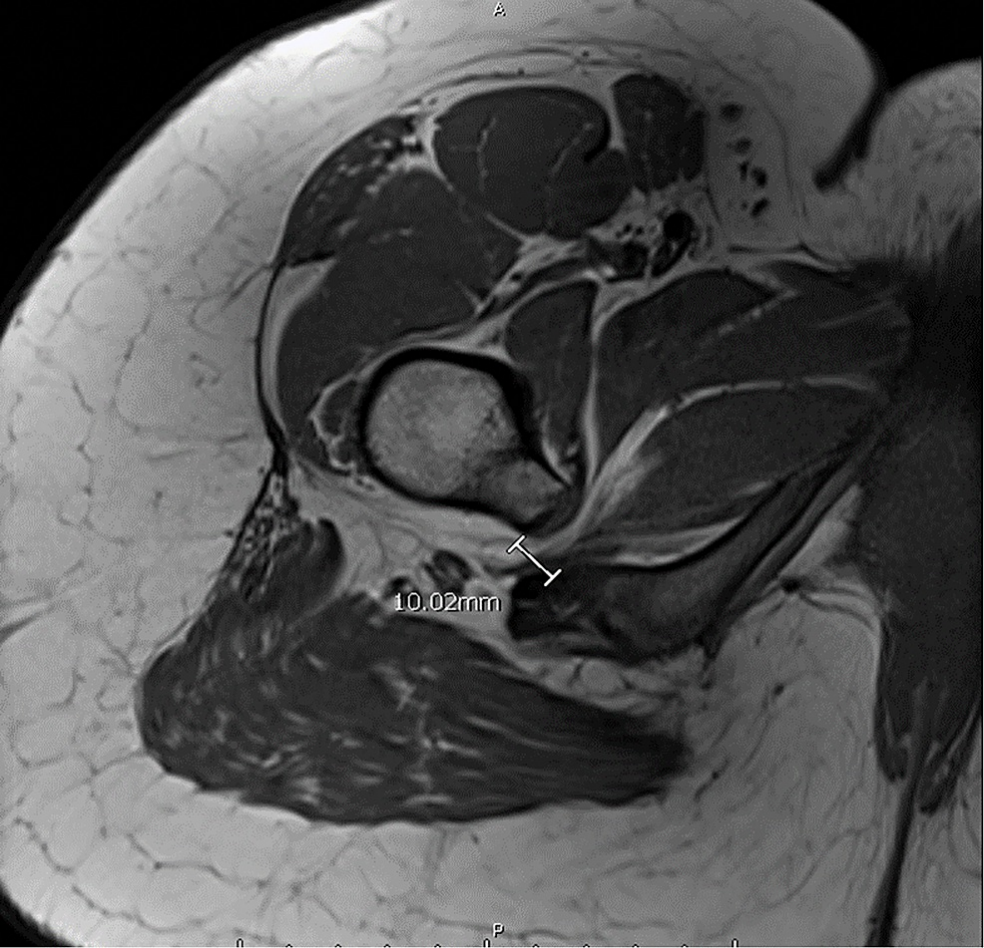
Ischiofemoral impingement is visualized on an axial T1 MRI. The distance between the lesser trochanter and the ischial tuberosity is approximately 10 mm, with evidence of fatty infiltration of the quadratus femoris muscle.

An MRI study was ordered and revealed left IFI with quadratus femoris edema (Figure 1). The ischiofemoral distance (IFD) is the shortest distance from the medial cortex of the lesser trochanter to the lateral cortex of the ischial tuberosity. The mean normal IFD for females is 18.6 + 8mm.3 This patient’s IFD was 10.02 mm. Abnormal IFD is defined as < 15 mm (sensitivity 77%, specificity 81%, accuracy 74%). The quadratus femoris space was measured as the shortest distance from the passage of the quadratus femoris between the posteromedial iliopsoas tendon and the superolateral hamstring tendons. The patient’s quadratus femoris space was measured as 4 mm on the left side. Abnormal quadratus femoris space is defined as < 10 mm (sensitivity 79%, specificity 74%, accuracy 77%) [1,5]. The patient’s working diagnosis was changed to IFI and she underwent CT-guided bilateral quadratus femoris muscle steroid injection. She reported that her pain level at the time of injection decreased by one point from a 5/10 to a 4/10 on the VAS.

The patient was subsequently referred for three courses of physical therapy over a one-year period. The first course consisted of two weeks of aquatic therapy followed by a stretching program (i.e., piriformis, gluteus maximus, iliopsoas, and quadriceps), while the second course included another four weeks of aquatics. As her symptoms did not improve, her physician ordered an MRI of the lumbar spine. This revealed additional findings of mild degenerative disc degeneration at L4-5 and L5-S1 levels. She was referred for a third course of physical therapy for continued aquatics training, with the goal of returning to pain-free running. She subsequently underwent a four-week running class for instruction in mid-foot running technique. After the three-month treatment period, she attempted a work-based physical fitness assessment; however, she had a recurrence of her initial pain complaints in her low back and the gluteal region as well as radiating pain with numbness on the left leg. A bone scan did not reveal any stress fractures.

One year following discharge from physical therapy, she was referred to the Interdisciplinary Pain Management Clinic (IPMC) for chronic hip pain. Her treatment plan included yoga, an anti-inflammatory diet, medical massage therapy, and referral to the physical therapist embedded in the IPMC. On presentation to physical therapy, the patient’s goal was to run and workout without pain and to successfully complete a work-based physical fitness test required for her to stay in her current profession. She wanted to explore all non-surgical treatment options including another trial of physical therapy.

Physical examination

Physical evaluation at the IPMC revealed that the patient’s pain was localized to the left posterolateral gluteal area. Digital pressure applied to the piriformis muscle belly reproduced her historical pain complaint greater than the pressure applied to the quadratus femoris. The patient’s pain was rated as 4/10 at rest and 6/10 during palpation on the VAS. A neurological clearing examination of L1-S1 revealed no dermatomal sensory changes or myotomal weakness in her bilateral lower extremities. The patient’s lumbar range of motion (ROM) was full and pain-free. Hip ROM was assessed passively with a goniometer and found to be within normal limits for all motions, although internal rotation was at the lower end of normal at 25 degrees.

Motion palpation tests

While standing, palpation revealed asymmetry in the patient’s pelvic landmarks with her posterior superior iliac spine (PSIS) and iliac crest elevated on the left side as compared to the right. The motion palpation tests utilized included the Forward Flexion test, Gillet’s test, and the Supine to Long Sit test. In the Forward Flexion test, the test was positive when the left PSIS on the involved side moved first when the patient bent forward (sensitivity 17%; specificity 79%) [6]. Gillet’s test was positive when the left PSIS on the involved side did not move inferiorly as the patient flexed her hip actively (sensitivity 8%; specificity 93%).6 The Supine to Long Sit test was positive for the potential contribution of the SIJ to apparent leg length discrepancy when the position of the left medial malleolus shortened in comparison to the right side as the patient sat up (sensitivity 44%; specificity 64%) [6].

Pain provocation tests

In the prone position, the pressure was applied from a posterior to an anterior direction to the spinous processes of L1 to L5. No pain or segmental hypomobility was noted, indicating no referred pain from the lumbar spine. In the supine position, a Thigh Thrust test was performed to assess for SI dysfunction. With the patient’s knee and hip flexed, the force was applied axially through the knee producing a shear force to the SI joint (sensitivity 88%; specificity 18%) [7]. The test was positive for pain and joint hypomobility on the left side and did provoke radiating pain to the left groin region. Finally, an active piriformis test was performed to detect muscle tightness and potential sciatic nerve entrapment (sensitivity 78%; specificity 80%) [8]. This test was positive for muscle tightness bilaterally and did reproduce her gluteal pain.

Ischiofemoral impingement tests

**Figure 2:**
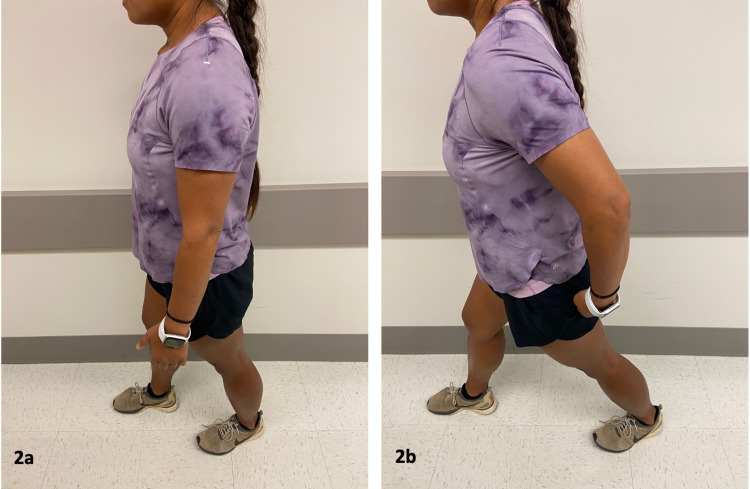
Long Stride Walking (LSW) Test. The LSW test is performed while standing. (a) When the patient walked with a shortened stride, the pain was not reproduced. (b) When the patient took a long step with the right foot thereby placing the left leg in extension. The test was positive when the pain just lateral to the ischial tuberosity was reproduced on the side that the hip was extended. *(Photograph: Newman, DP. Long Stride Walking Test. Reproduced with permission of the author, 2021).*

**Figure 3:**
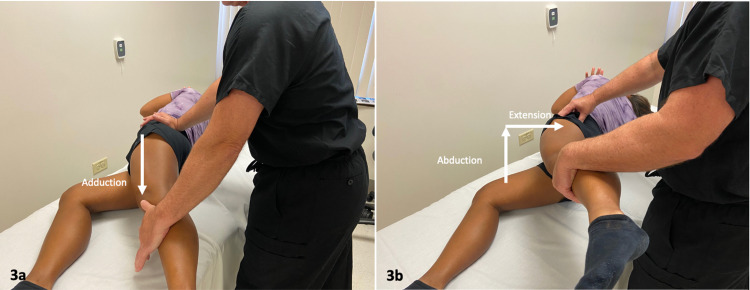
Ischiofemoral Impingement Test. The ischiofemoral impingement test is performed with the patient lying on her right side. The affected hip is passively adducted. The test was positive when the index pain was reproduced in this position (a), and pain resolved when the hip was then moved into hip extension and then abducted (b). *(Photograph: Newman, DP. Ischiofemoral Impingement Test. Reproduced with permission of author, 2021).*

Two special orthopedic tests to detect possible IFI were applied; the long-stride walking (LSW) test (Figures 2a, 2b) and the ischiofemoral impingement test (Figures 3a, 3b) [9]. The sensitivity and specificity of each test are 94% and 85%, and 82% and 85%, respectively. Both tests were positive for this patient on the left side. While there were MRI findings for impingement on the right side, these tests were both negative.

Diagnosis/prognosis

The differential diagnosis for this patient’s symptoms on initial evaluation at the time of presentation to the IPMC included low back pain, SI joint dysfunction, IFI, and piriformis syndrome. While she did have degenerative changes at L4-S1 on x-ray and MRI, these findings were mild and provocation testing to the facet joints did not reproduce her symptoms; therefore, low back pain was less likely. SI joint dysfunction was considered based on the multiple positive motion palpation and provocation tests. The biomechanical fault at the SI joint may have been contributing to increased loading upon the piriformis and quadratus femoris. A diagnosis of IFI correlated with the MRI findings and positive IFI test; however, this diagnosis was equivocal given the poor response to a diagnostic and therapeutic corticosteroid injection to the quadratus femoris. Kim, Yoon, and Yoon reported a 50% reduction in pain in 14 patients following quadratus femoris injections while Volokhina and Dang noted complete resolution in a patient following an injection for IFI [10,11]. In our case, the patient reported a 1-point reduction in pain over a two-month period. Therefore, piriformis tightness in conjunction with SI joint dysfunction was selected as the working diagnosis.

The prognosis for full resolution of symptoms was poor given the chronicity of symptoms and the poor response to previous conservative and interventional measures. Additionally, the patient’s goals of jogging two miles under 24 minutes without pain, which was an occupational requirement, appeared to be unrealistic as her previous run-focused physical therapy regimen was unsuccessful.

Intervention

To validate the working diagnosis and identify contributing pain generators, an intervention-based diagnostic approach was utilized. First, biomechanical faults remote to the area of pain would be addressed. Surrounding tissues to include the piriformis would be treated to determine if this was the primary pain generator. After the interventions, the patient would test the treatment efficacy by climbing stairs immediately after treatment and then jogging later in the day. She reported that her baseline pain was reproduced after climbing two flights of stairs and jogging the length of three light posts spaced 30 meters apart near her home (90 meters).

**Figure 4:**
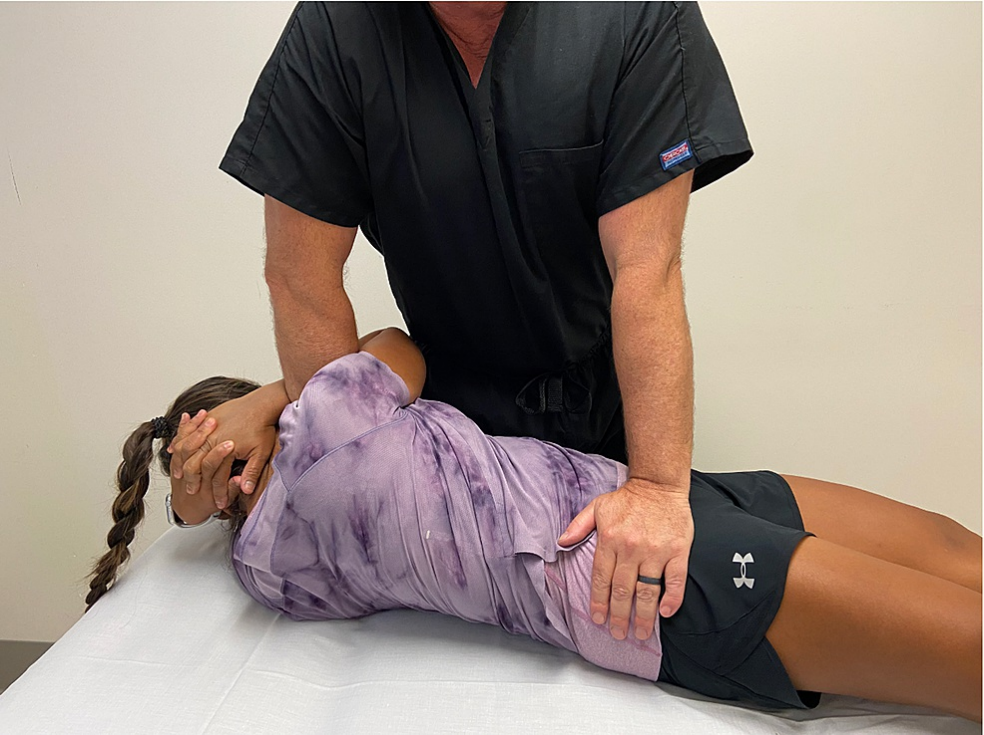
Sacroiliac Joint Manipulation Technique. With the patient in supine, the provider passively side bends the patient to the right and then rotates the trunk to the left locking down the lumbar spine.  The provider pushes the right ilium into a posterior direction until a barrier is felt.  As the patient exhales, the provider imparts a high velocity, low amplitude force downwards. *(Photograph: Newman, DP. Sacroiliac Joint Manipulation Technique. Reproduced with permission of the author, 2021).*

**Figure 5:**
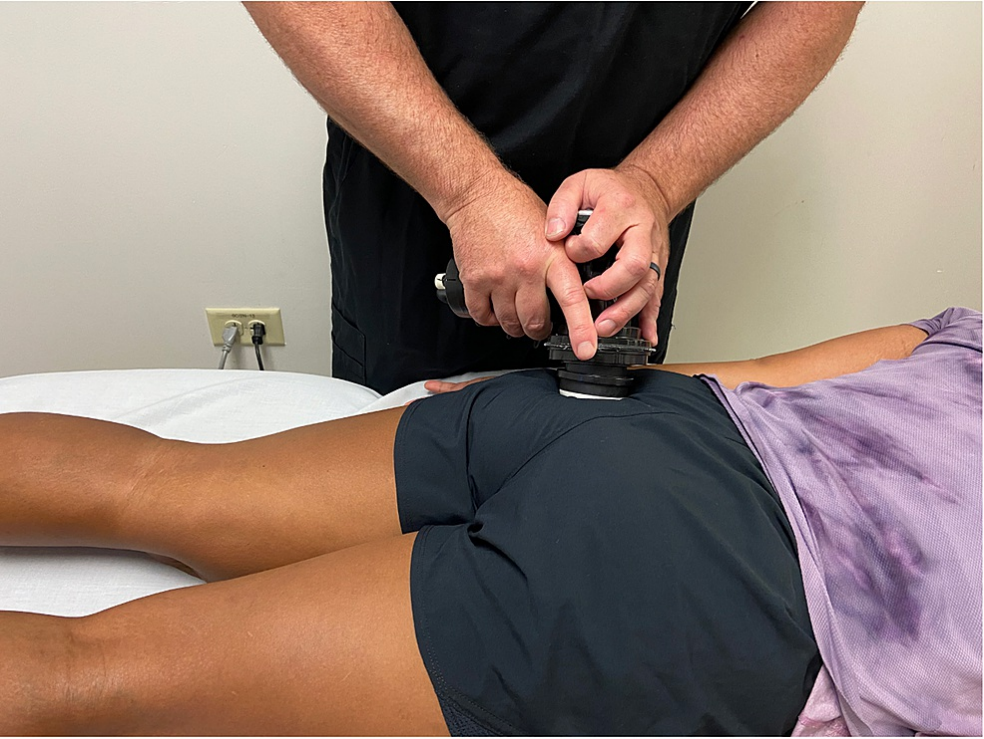
Instrument Assisted Deep Tissue Mobilization to the Piriformis Muscle. While the patient was prone, the Vibracussor© was applied over the piriformis. The tool was moved superiorly and inferiorly along the course of the muscle just medial to the insertion at the greater trochanter to just lateral to the sacrum. *(Photograph: Newman, DP. Instrument Assisted Deep Tissue Mobilization to the Piriformis Muscle. Reproduced with permission of the author, 2021).*

After the physical examination, the patient underwent a common osteopathic manipulation technique (OMT) directed at the left ilium to address the SI joint dysfunction (Figure 4). After the maneuver, the patient reported no groin pain during a repeat Thigh Thrust test. To identify the impact of piriformis tightness and the effect of stretching on achieving treatment success, instrument-assisted deep tissue mobilization (IASTM) using the VibraCussor® (IMPAC Inc., Salem, OR) was directed at each piriformis muscle for five minutes (Figure 5). Immediately after treatment, she climbed three flights of stairs at which point her posterolateral gluteal pain was reproduced. She was instructed to go jogging that evening. The patient used the number of light posts, spaced 30 meters apart, that she could jog past until the onset of pain as an objective way to assess jogging tolerance. She would also assess functional progress based on the number of flights climbed as climbing stairs was a work-related requirement.

The patient was started on a home exercise program consisting of piriformis stretching and lumbopelvic stabilization exercises directed at improving muscle endurance of the abdominals, hamstrings, and hip adductors. These muscles are critical for creating a force couple to posteriorly tilt the pelvis thereby stabilizing the pelvis and elongating the hip flexors [12]. The patient was asked to follow up one week later at which point a more comprehensive plan of care would be developed based on the initial treatment result.

At the second visit (one week after initial evaluation), the patient reported no baseline pain at rest. She ran the distance of five light posts (150 meters) and climbed four flights of stairs until reproduction of pain (5/10 level). Her physical examination revealed asymmetrical pelvic landmarks and positive motion palpation and pain provocation tests for left-sided SIJ joint dysfunction; however, both of the IFI tests were negative. Pain with digital pressure was reproduced over the piriformis muscle belly, but not the quadratus femoris. Given these findings, it appeared that the quadratus femoris edema in the presence of decreased IFD may have been an incidental finding. Her primary diagnosis was piriformis syndrome.

The plan of care would include manual therapy, stretching, strengthening, IASTM, and a progressing jogging program (Table 1). An impairment-based rehabilitation program was designed to leverage OMT and IASTM with a home-based exercise program. While the treatment duration to meet her jogging goal was initially estimated as eight weeks, the actual time period for meeting the patient goals was 16 weeks.

**Table 1:** Overview of Interventions Applied and Patient Response per Visit. (Sixth-eighth visits not included as there was no significant change in symptoms).

Visit (Period after initial evaluation)	Patient Pain Presentation	Objective Findings	Intervention	Patient Response
1	Pain localized to area of the right piriformis greater than the quadratus femoris. Pain exacerbated with jogging and climbing stairs.	Pelvic asymmetry with positive SI joint dysfunction tests. Positive Thigh Thrust test with additional left groin pain. Pain reproduced with digital pressure to the piriformis and quadratus femoris muscle bellies. Positive Active Piriformis Test with gluteal pain reproduction. Positive IFI and LSW tests.	Left SI joint OMT. IASTM to bilateral piriformis muscles for 5 minutes. Instruction in piriformis stretching and lumbopelvic stabilization exercises.	Increased stair climbing tolerance (4 flights completed compared to pain elicited at 2 flights). Climbed 4 flights of stairs until pain provocation (5/10 pain level).
2 (1 week)	No baseline posterolateral hip pain upon follow-up. Pain exacerbated with jogging and climbing stairs. Ran 5 light posts (150 meters) until onset of pain (5/10 pain level).	Pelvic asymmetry with positive SI joint dysfunction tests. Pain reproduced with digital pressure to the piriformis, but NOT the quadratus femoris. Negative IFI and LSW tests. Treadmill jogging at 5 mph / 0% incline for 3:30 minutes until pain reproduced.	Left SI joint OMT. IASTM to the left piriformis for 5 minutes.	Climbed 7 flights of stairs without pain. Treadmill jogging for 4:20 minutes until pain reproduction.
3 (2 weeks)	No pain at rest. No pain with jogging on the beach. Short period of pain following sprint / walk on track for 4 miles. 5/10 pain reproduced with running length of 5 light posts but resolved after 4-5 minutes of rest.	Symmetric pelvic landmarks. Negative SI joint dysfunction tests. Negative IFI and LSW tests. Pain with digital pressure over the quadratus and piriformis muscle bellies. Groin pain reproduced with assessment of iliopsoas tightness on the left side. Positive Active Piriformis Test. Treadmill jogging for 3 minutes until onset of pain.	IASTM to the left piriformis for 5 minutes. ART and manual stretching of the left iliopsoas. Added iliopsoas stretching to the home program.	Decreased pain to palpation of the quadratus femoris and piriformis following ART to the iliopsoas. Treadmill jogging for 4:00 minutes until pain reproduction.
4 (3 weeks)	Pain flare with carrying boxes up two flights of stairs (6/10) for one hour. Ran length of 7 light poles (210 meters) with 5/10 pain but decreased to 1/10 pain following piriformis stretching.	Piriformis tightness.	IASTM to the left piriformis.	Treadmill jogging for 6:44 minutes until pain reproduction.
5 (7 weeks)	No increase in pain with increased activity. 5 hours of muscle soreness after a 1.6-mile hike. No pain with prolonged walking or stair climbing. Pain with jogging the length of 5 light posts (150 meters).	Piriformis tightness.	IASTM to the left piriformis.	Treadmill jogging of 6:04 minutes before pain reproduction.
9 (4 months)	No pain upon examination. Ran 2 miles in 22:30 minutes without pain.	Benign examination.	Discharge from IPMC.	

Prior to treatment, jogging tolerance was tested by jogging on a treadmill (5 mph/0% grade) to the point of pain reproduction. The pain was reproduced after 3:30 minutes. The left SI joint OMT and IASTM were repeated to the left piriformis only as the patient did not have pain on the right side. After treatment, she was able to climb seven flights of stairs without pain reproduction. To test jogging tolerance, she ran on a treadmill at the same pace and grade for 4:20 minutes until the posterolateral hip pain was reproduced. She would continue the prescribed exercise program emphasizing piriformis stretching, both before and after working out and jogging. She was instructed to keep a pain journal identifying what physical activity she participated in, pain response, and time of pain resolution. She would follow up in one week.

At the 3rd visit, the patient reported no pain at rest. Over the one-week period, she jogged on the beach pain-free and completed an obstacle course with only mild soreness. She performed sprint/walk intervals on a track for a total distance of four miles in 42 minutes. Her original 5/10 pain returned but quickly resolved. The next day she ran the length of five light poles with 5/10 pain which lasted four to five minutes after stopping. On exam, there was tenderness to palpation over the left piriformis and quadratus femoris muscle bellies. The Thigh Thrust test and both IFI tests were negative. The patient’s left-sided groin pain was reproduced with an assessment of the iliopsoas muscle length, and the piriformis test was positive. She ran on the treadmill for three minutes at which point her pain level increased to a 5/10.

**Figure 6:**
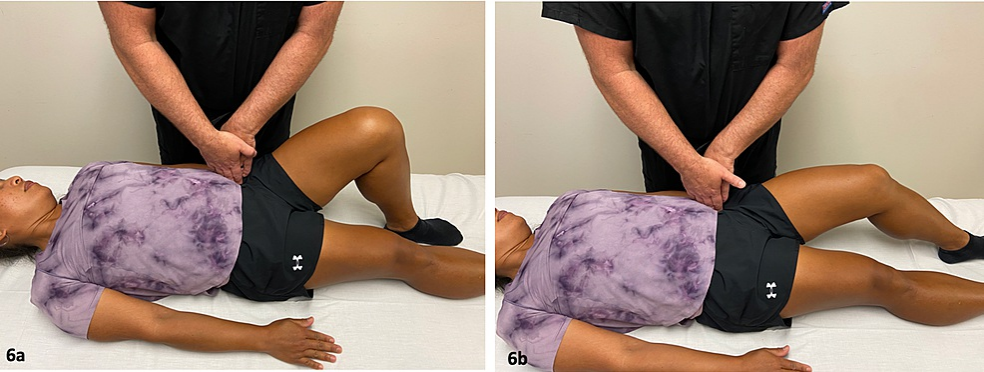
Iliopsoas Active Release Technique. While the patient was supine with her hip flexed to 90 degrees, the provider applied digital pressure inferiorly and laterally pinning the iliacus muscle against the inside of the pelvic rim (a). The patient then actively extended her leg fully (b). The provider released the pressure and the patient returned her leg to the starting position. This was repeated five times. *(Photograph: Newman, DP. Iliopsoas Active Release Technique. Reproduced with permission of the author, 2021).*

Treatment consisted of IASTM to the left piriformis and active release technique (ART) followed by manual stretching of the iliopsoas (Figures 6a, 6b). Piriformis IASTM reduced pain to palpation over the quadratus femoris and iliopsoas ART reduced pain to palpation over the quadratus femoris and piriformis. She then ran on the treadmill for four minutes at which point her pain was reproduced. While the mechanism of response is confounding, the authors posit that iliopsoas tightness was contributing to increased loading of the piriformis and quadratus femoris muscles while jogging. Her home exercise program included iliopsoas stretching.

At the fourth visit, the patient was able to walk/sprint two miles without pain. She reported a period of 6/10 pain for an hour after carrying heavy boxes up two flights of stairs. The following day, she ran the length of seven light poles (210 meters). While her pain increased to a 5/10, it decreased to a 1/10 after piriformis stretching. Her exam revealed only piriformis tightness. After IASTM to the left piriformis, she was able to jog 6:44 minutes at 5 mph until the return of symptoms. The patient was instructed to continue her exercise program and follow-up in one month.

One month later (fifth visit), the patient endorsed no increase in pain with increased activity levels. She completed a 1.6-mile hike with a 171-meter elevation gain. She had soreness for five hours, compared to her baseline of 12-24 hours of pain after physical activity. She did not have pain with prolonged walking or stairs. She continued to experience pain with jogging the distance of five light poles (150 meters). Her physical exam only demonstrated pain reproduction with the piriformis test. No pain was noted along the quadratus femoris with deep palpation. After IASTM to the piriformis, she ran for 6:04 minutes on the treadmill with 3/10 pain reproduction.

The patient was instructed to follow up every two weeks (6th through 8th visits) for treatment which consisted of IASTM and manual stretching to the left piriformis to complement her home exercise program. By the ninth visit, the patient completed her physical fitness assessment by jogging two miles in 22:30 minutes. She did not report any recurrence in pain. The patient was subsequently discharged from the IPMC.

## Discussion

In patients presenting with deep gluteal pain, the differential can be extensive and require an exhaustive workup that takes time and requires expensive diagnostic testing. Carro and colleagues (2016), describe several diagnoses encompassed within the umbrella of the deep gluteal syndrome (DGS) [13]. These conditions include piriformis syndrome, IFI, Gemelli-obturator internus syndrome, and hamstring pathology impacting the sciatic nerve. There is significant overlap in signs and symptoms experienced by patients with these diagnoses to include pain and/or dysesthesias in the buttocks, posterior thigh, hip, and potentially radicular symptoms due to irritation of the sciatic nerve.

While commonly reported as a pathoanatomic cause of sciatica, piriformis syndrome is not well understood and difficult to accurately diagnose given no gold standard test [14]. An MRI can provide diagnostic clarity, with piriformis muscle hypertrophy visualized on the involved side [15]. Magnetic resonance neurography offers good diagnostic efficacy (sensitivity of 64%, specificity of 93%) and magnetic resonance-guided anesthetic injections to the piriformis have been shown to provide short-term benefit suggesting more of a diagnostic utility than therapeutic [16].

Fishman and colleagues (2016) provide justification for identifying patients with piriformis syndrome in the presence of a prolonged H-reflex (three standard deviations) while performing hip flexion, adduction, and internal rotation (sensitivity of 88%, specificity of 83%) [17]. Those patients selected were treated with a combination of a piriformis injection and physical therapy. At 10 months, 79% of patients experienced at least 50% improvement in pain and functional disability.

In our case, the patient’s MRI did not demonstrate any pathology related to the piriformis; instead, the patient met the diagnostic criteria for IFI with decreased ischiofemoral space and quadratus femoris edema. Three courses of physical therapy over a one-year period based on the diagnosis of IFI were unsuccessful. Additionally, the corticosteroid injection to the quadratus femoris provided minimal relief; therefore, the diagnostic utility of a negative response would suggest another pain generator.

The authors posit that most likely, two DGS conditions were present concomitantly. The patient did have a positive IFI test and LSW test and pain produced with digital pressure to the quadratus femoris upon presentation to the IPMC PT; however, both special tests were negative, and the pain was not reproduced over the quadratus femoris after her first treatment (first follow-up visit). The initial treatment was directed at the SI joint asymmetry and piriformis muscle tightness. This would suggest that either or both of these impairments may contribute to increased loading of the quadratus femoris muscle. Conversely, the IFI may have predisposed the piriformis and sciatic nerve to injury. It is also possible that the IFI findings on MRI were incidental. This may suggest that MRIs may be overprescribed and utilized in lieu of a comprehensive physical examination. While re-imaging of the quadratus femoris upon completion of her treatment could be confirmatory, we deferred this option given its limited clinical value.

Conservative management of patients presenting with gluteal pain due to piriformis-mediated entrapment of the sciatic nerve or piriformis tightness generating gluteal pain can be successfully performed through a multimodal treatment program previously described by Newman and colleagues [18]. The initial treatment was designed to identify potential impairments of the SI joint and surrounding tissues. The SI joint dysfunction and piriformis tightness was addressed initially. After the treatment, response was determined by having the patient climb stairs, results of which did improve. Unfortunately, it is difficult to distinguish which impairment was most responsible for the initial outcome, and diagnostic accuracy may have been improved if the patient climbed the stairs after each treatment individually.

At the first reassessment, the IFI tests were negative. On the third visit, digital pressure to the quadratus femoris did reproduce pain, and pain was reproduced in the groin region with assessment of the iliopsoas muscle length. After ART was applied to the iliopsoas muscle, there was less pain produced with digital pressure applied to the piriformis and quadratus femoris. A potential explanation for the interaction of the iliopsoas with the piriformis and quadratus femoris may lie in the biomechanical role of these muscles to stabilize the pelvis and hip. Injury of one muscle may result in compensatory overworking of the others [19].

The patient’s presentation on serial reassessment (fourth through eighth visits) demonstrated only piriformis tightness. After IASTM to the piriformis muscle belly, the patient would reassess jogging tolerance on a treadmill. The patient’s baseline duration at 5 mph until pain onset was 3:30 minutes (second visit). This duration would always be greater than baseline after IASTM. The patient noted mitigation in pain following jogging by stretching the piriformis. Improved jogging tolerance and faster recovery serve to validate the importance of piriformis lengthening on running tolerance.

This case study has several limitations. The results may not be generalizable to all patients presenting with both IFI and piriformis syndrome. As discussed above, it is not certain that the IFI was an incidental finding given the fact that the treatment directed at addressing the SI joint mechanics and piriformis syndrome resulted in negative IFI tests. The patient’s initial physical therapy treatment included iliopsoas and piriformis muscle stretching but did not include manipulation of the SI joint. Manipulation of the SI joint may be the key to an effective treatment program.

Additionally, the conservative management of IFI may not require addressing all of the muscles surrounding the hip but may instead be targeted solely to iliopsoas and piriformis muscle tightness [19]. Future case-controlled studies are necessary to determine if piriformis tightness is more prevalent in patients diagnosed with IFI based on MRI findings. If this is true, conservative management may be better optimized and enhance selectivity for quadratus femoris injections or surgical decompression of the ischiofemoral space.

## Conclusions

Hip pain is a common problem, especially in young adults. The cause of the pain can be difficult to diagnose given the wide differential diagnoses. IFI is a relatively new diagnosis with specific radiographic findings. In this case, the patient underwent several courses of physical therapy despite a negative confirmatory quadratus femoris injection. The patient’s diagnosis was expanded to include piriformis syndrome with the patient ultimately achieving complete resolution of pain. This case highlights the need to consider the possibility that a patient may have two different diagnoses at the same time and that treatment needs to be adjusted when initial programs fail to achieve the desired result.
